# An expandable synthetic library of human paired antibody sequences

**DOI:** 10.1371/journal.pcbi.1012932

**Published:** 2025-04-21

**Authors:** Toma M. Marinov, Perry T. Wasdin, Gwen Jordaan, Alexis K. Janke, Alexandra A. Abu-Shmais, Ivelin S. Georgiev

**Affiliations:** 1 Vanderbilt Center for Antibody Therapeutics, Vanderbilt University Medical Center, Nashville, Tennessee, United States of America; 2 Center for Computational Microbiology and Immunology, Vanderbilt University Medical Center, Nashville, Tennessee, United States of America; 3 Vanderbilt Institute for Infection, Immunology and Inflammation, Vanderbilt University Medical Center, Nashville, Tennessee, United States of America; 4 Department of Pathology, Microbiology, and Immunology, Vanderbilt University Medical Center, Nashville, Tennessee, United States of America; 5 Department of Computer Science, Vanderbilt University, Nashville, Tennessee, United States of America; 6 Department of Biomedical Informatics, Vanderbilt University, Nashville, Tennessee, United States of America; 7 Department of Chemical and Biomolecular Engineering, Vanderbilt University, Nashville,Tenessee, United States of America; 8 Department of Biochemistry, Vanderbilt University, Nashville, Tennessee, United States of America; 9 Center for Structural Biology, Vanderbilt University, Nashville, Tennessee, United States of America; Fred Hutchinson Cancer Research Center, UNITED STATES OF AMERICA

## Abstract

The potential diversity in the global repertoire of human antibody sequences is currently not well understood due to the limited existing paired antibody heavy-light chain sequence data that has been hindered by the low throughput and high costs of current single-cell sequencing methods. Here, we report IgHuAb, a large language model for high-throughput generation of paired human antibody sequences. Using IgHuAb, we created SynAbLib, a synthetic human antibody library that mimics population-level features of naturally occurring human antibody sequences, yet is associated with significantly greater diversity in sequence space. Further, experimental validation of a diverse set of antibodies from SynAbLib showed robust expression yields. IgHuAb and SynAbLib provide a readily expandable platform for human monoclonal antibody generation that can be efficiently mined for antibody sequences with target properties.

## Author summary

Antibodies are proteins secreted by B-cells. They help the immune system respond against foreign pathogens by binding to them, directly blocking their ability to infect, marking them for destruction by other immune cells, and/or activating additional immune responses. This makes antibodies excellent candidates for the development of novel therapeutics. However, antibody discovery and development are limited by the fact that traditionally antibodies are isolated from biological samples, making the process difficult, expensive and time consuming. To address this challenge, novel developments in computational approaches for antibody discovery can present effective and efficient alternative strategies to traditional antibody discovery. Here, we present a new antibody large language model algorithm, IgHuAb, that is capable of efficiently generating human antibodies that mimic the protein sequence features of naturally occurring antibodies. We applied IgHuAb to create a synthetic library, SynAbLib of over 1 million human antibody sequences, that is readily expandable to virtually unlimited diversity. IgHuAb and SynAbLib can be used for the discovery and/or optimization of new antibodies for medical treatment, for gaining novel insights into the fundamental characteristics of human antibody sequences, or to benchmark/train other antibody AI algorithms.

## Introduction

Monoclonal antibodies are an effective therapeutic modality against a wide range of diseases, including infectious disease, cancer, autoimmunity, and others [1,2]. Antibodies are also important as diagnostic and research reagents, and can serve as templates for the development of effective vaccines. Monoclonal antibodies are the secreted form of the B cell receptor, which, in humans, consists of a pairing of a heavy chain (HC) and a light chain (LC) protein. HC-LC pairing is one of the mechanisms that enables diversification of the antibody repertoire, along with germline gene recombination and somatic hypermutation in each of the HC and LC. Because of these different antibody diversification mechanisms, the potential space of antibody sequences is exceptionally large [3,4]. While advances in single-cell technologies have drastically improved the throughput of antibody discovery methods for characterizing the antibody sequence space [5,6], such methods for data generation are resource intensive and difficult to scale, leading to only limited numbers of paired HC-LC antibody sequences that are recovered per experiment [7,8].

With recent advances in artificial intelligence (AI) and computational biology, the feasibility and accuracy of modeling protein sequence and structure has increased immensely, with effective applications to drug, vaccine, and biosensor design, among many others [9,10]. In the realm of antibodies, optimization of existing antibody candidates through targeted redesign of the initial sequence/structure of a traditionally sourced antibody has been accomplished [11,12]. AI approaches are particularly suited to identifying associations between inputs and outcomes, without necessarily explicitly deciphering the complex underlying relationships. Recently, large language models (LLM), which have been successfully applied to numerous areas outside of language processing [10,13], have been shown to effectively capture the fundamental rules of protein sequence and function by viewing proteins as a string (a sequence of characters). Such models have also recently been adapted to generate novel antibody sequences [14–18]. Given the significance of native antibody HC-LC pairing, the development of AI methods for paired HC-LC sequence generation is therefore well-motivated and an important gap in current technological capabilities.

Here we present IgHuAb, an LLM-based approach for the generation of paired HC-LC sequences for human antibodies. IgHuAb can be applied to the creation of synthetic human antibody libraries of size and diversity significantly beyond what can be obtained with current single cell sequencing technologies. Such synthetic antibody libraries will be enabling in a variety of settings, including as a resource for antibody discovery and optimization, as a training dataset for AI algorithms, and to provide novel insights into the fundamental characteristics of human antibody sequences. Using IgHuAb, we created SynAbLib, a synthetic human antibody library that is easily generalizable and expandable. Computational assessment and experimental validation of SynAbLib highlight the potential of this dataset as a resource for antibody science.

## Results

**Fig 1:**
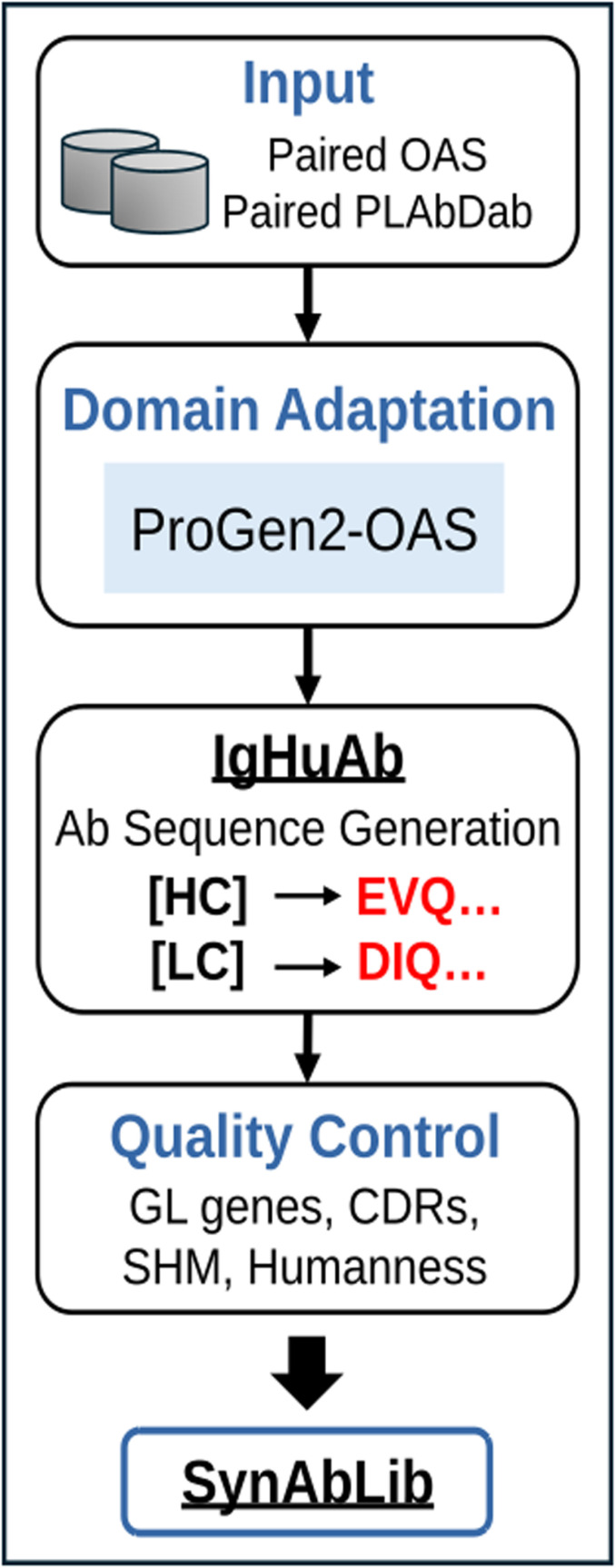
Schematic Diagram of IgHuAb and SynAbLib. The curated data contains paired human antibody sequences from the Paired OAS and the PLAbDab databases. For domain adaptation fine-tuning, the ProGen2-OAS model was used. After fine-tuning, the IgHuAb model was used to generate the paired human antibody sequences. The synthetic paired antibody library SynAbLib consists of IgHuAb-generated sequences that passed quality control: human germline gene assignment, CDR assignment, mutational load and humanness score estimation.

IgHuAb was developed by fine-tuning a pretrained protein LLM for the task of generating paired HC-LC antibody variable sequences (**Fig 1**). ProGen2 [16], a suite of general protein LLMs, includes a version which was exclusively trained on unpaired heavy and light chain antibody sequences (ProGen2-OAS), but this model is incapable of generating paired antibody sequences. We therefore aimed to extend the capabilities of ProGen2-OAS by fine-tuning on a dataset of 430,000 paired, human antibody sequences curated from the PLAbDab (Patent and Literature Antibody Database) [19] and paired-OAS (Observed Antibody Space)(*8*) databases, allowing the model to learn the relationships underlying native HC-LC pairing in human antibodies. During fine-tuning, the heavy and light chain sequences were denoted by introducing new tokens, [HC] and [LC], enabling the model to be prompted with either token to generate novel paired antibody sequences (Methods); we will refer to these as the [HC] and [LC] datasets. After training, IgHuAb was used to generate a large synthetic library of paired sequences which recapitulates the sequence feature distributions captured by experimental databases, while extending the sequence space to provide novel antibody sequences for downstream analysis, model development, and antibody discovery efforts.

**Fig 2:**
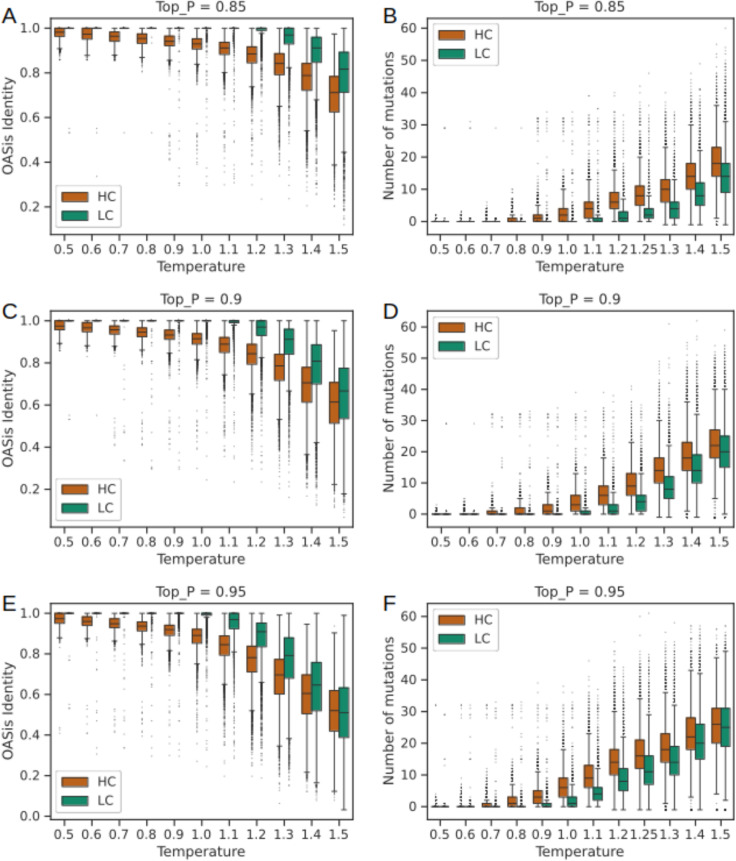
OASis Identity Score and Number of Mutations of the IgHuAb generated HC (in red) and LC (in green) sequences for top P =0.85, 0.9, 0.95 and T=0.5 - 1.5. (A) OASis Identity Score: top P=0.85 and T=0.5 - 1.5, (B) Number of Mutations: top P=0.85 and T=0.5 - 1.5, (C) OASis Identity Score: top P=0.9 and T=0.5 - 1.5, (D) Number of Mutations: top P=0.9 and T=0.5 - 1.5, (E) OASis Identity Score: top P=0.95 and T=0.5 - 1.5, (F) Number of Mutations: top P=0.95 and T=0.5 – 1.5.

We first aimed to establish the ability of IgHuAb to generate antibody sequences that capture the overall characteristics of human paired antibody sequences that are found in nature. Towards this goal, we explored the effects of the hyperparameters Top_P and temperature, which control the randomness and diversity of the generated sequences. For each Top_P [0.85, 0.9, 0.95] and temperature T [0.5, 0.6,…1.5] we generated a dataset of 10,000 sequence pairs, yielding a total of 330,000 HC-LC pairs which were then aligned to human germlines to calculate the number of mutations in the V genes (up to and including FWR3) for the HC and LC. To evaluate whether the IgHuAb-generated antibody HC-LC pairs resembled human antibody sequences, we utilized BioPhi OASis [20], an open-source platform for scoring antibody ‘humanness’ (**Fig 2**). For all values of Top_P, with an increase in temperature, we observed an increase in the number of mutations in the generated HC-LC pairs and a decrease in the OASis identity scores.

**Fig 3:**
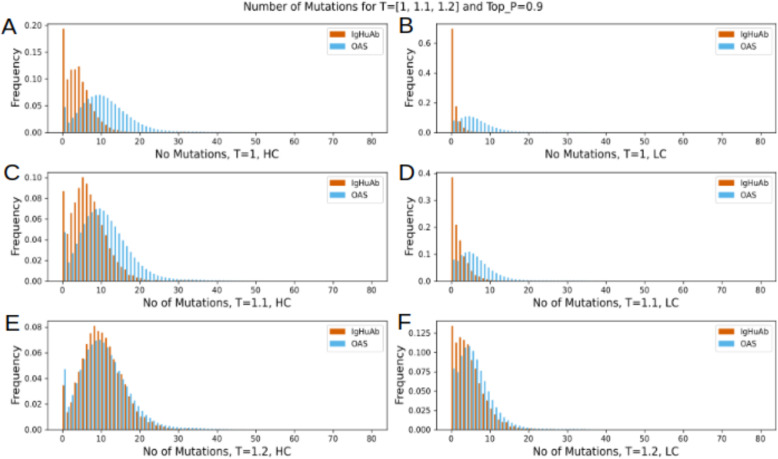
Number of Mutations (up to CDR3) of the IgHuAb generated HC and LC for top P =0.9 and T=1, 1.1 and 1.2 vs Number of Mutations of OAS HC and LC (in blue). (A) Number of Mutations of HC: top P=0.9 and T=1, (B) Number of Mutations of LC: top P=0.9 and T=1, (C) Number of Mutations of HC: top P=0.9 and T=1.1, (D) Number of Mutations of LC: top P=0.9 and T=1.1,(E) Number of Mutations of HC: top P=0.9 and T=1.2. OAS(mean 10.24, std 6.36), IgHuAb(mean 9.86, std 5.35), (F) Number of Mutations of LC: top P=0.9 and T=1.2 OAS(mean 5.72, std 4.69), IgHuAb(mean 4.42, std 3.86).

We next compared the number of mutations observed for the IgHuAb-generated sequences to the number of mutations observed in the OAS, for different temperatures and Top_P=0.9 (**Fig 3**). For lower temperatures, OAS antibody sequences exhibited greater numbers of mutations (Fig 3A-D). However, for T=1.2, the IgHuAb-generated sequences closely matched the mutation (up to and including FWR3) distribution of the OAS data: OAS HCs (mean 10.24, std 6.38) vs. IgHuAb-generated HCs (mean 9.86, std 5.35) (**Fig 3E**); OAS LCs (mean 5.72, std 4.69) vs. IgHuAb-generated LCs (mean 4.42, std 3.86) (**Fig 3F**). We therefore selected the Top_P=0.9 and T=1.2 hyperparameter values as default for the IGHuAb sequence generation.

**Fig 4:**
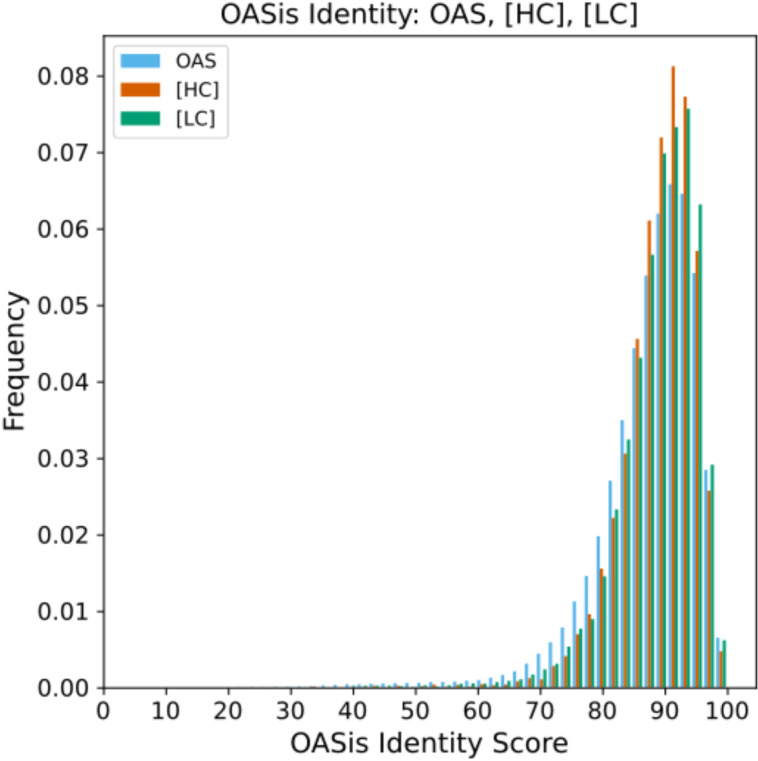
Distribution of OASis BioPhi Identity Scores. OAS(in blue) (mean 87.42, std 8.30), [HC] prompt (in red)(mean 89.00, std 5.92), [LC] prompt (in green)(mean 88.86, std 6.53).

We also found that IgHuAb-generated antibodies had a high degree of “humanness” by comparing OASis identity scores of the training dataset of OAS human antibodies (mean 87.42, std 8.30) to the [HC] prompt generated antibodies (mean 89.00, std 5.92) and the [LC] prompt generated antibodies (mean 88.86, std 6.53) (**Fig 4**).

**Fig 5:**
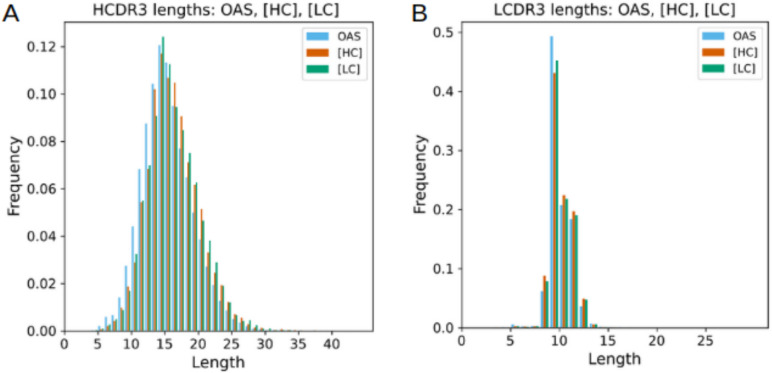
Comparison of CDR3 Length Distributions in IgHuAb and OAS. (A) Heavy Chain CDR3 Length Distribution: Paired OAS in blue(mean 14.99, std 3.76), generated with the [HC] prompt in red(mean 15.74, std 3.82), generated with the [LC] prompt in green(mean 15.76, std 3.87(B)Light Chain CDR3 Length Distribution: Paired OAS in blue(mean 9.63, std 1.11), generated with the [HC] prompt in red(mean 9.68, std 1.12), generated with the [LC] prompt in green(mean 9.66, std 1.11).

Next, for each of the [HC], [LC], and OAS datasets, we compared the distributions of the lengths of complementarity determining region 3 for both the heavy (CDRH3) and light (CDRL3) chain, as these distributions are well-established for naturally occurring antibodies. Importantly, we observed that the CDRH3 and CDRL3 lengths were similar for all three antibody sets (**Fig 5**). The CDRH3 length of OAS antibodies (mean 14.99, std 3.79), was comparable to the CDRH3 lengths for the [HC] prompt antibodies (mean 15.74, std 3.82) and the [LC] prompt antibodies (mean 15.76, std 3.87) (**Fig 5A**). Similarly, the CDRL3 length (mean 9.63, std 1.11) for the OAS antibodies was comparable to the CDRL3 lengths for the [HC] prompt antibodies (mean 9.68, std 1.12) and [LC] prompt antibodies (mean 9.66, std 1.11) (**Fig 5B**).

**Fig 6:**
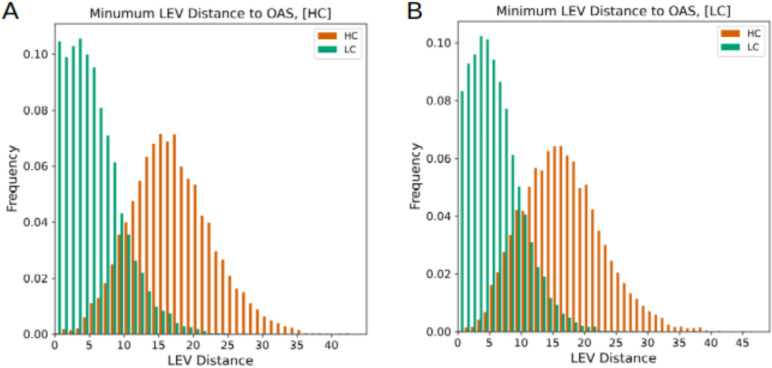
Distribution of Levenshtein Distances to OAS. (A) Minimum Levenshtein Distance to OAS, [HC] prompt: distance of generated heavy chains to heavy chains in OAS in red (mean 16.48, std 5.94), distance of generated light chains to light chains in OAS in green (mean 5.09, std 4.04), (B) Minimum Levenshtein Distance to OAS, [LC] prompt: distance of generated heavy chains to heavy chains in OAS in red (mean 16.12, std 6.36), distance of generated light chains to light chains in OAS in green (mean 5.43, std 4.11).

Next, we sought to determine if IgHuAb was capable of generating diverse antibody sequences, or whether it simply recaptured the close vicinity around existing antibody sequences. To ensure that IgHuAb did not simply reproduce training data sequences, we compared the minimum Levenshtein distance of each IgHuAb-generated HC and LC to each OAS training data HC and LC, respectively (**Fig 6**). Notably, for both the [HC] prompt and [LC] prompt generated antibodies, the heavy chain sequences exhibited a broad distribution of differences from OAS antibodies. As could be expected, based on the established lower diversity in light chain sequences [7], the generated antibodies were substantially closer to OAS antibody light chain sequences. For antibody pairs generated with the [HC] prompt, the minimum Levenshtein distance to OAS had a mean value of 16.48 (std 5.94) for HCs and a mean value of 5.09 (std 4.04) for LCs. For antibody pairs generated with the [LC] prompt, the minimum Levenshtein distance to OAS had a mean value of 16.12 (std 6.36) for HCs and a mean value of 5.43 (std 4.11) for LCs.

These results indicated that IgHuAb is capable of generating paired antibody sequences with lengths, humanness, and diversity that reproduce distributions of naturally occurring human antibodies at the population level. Additionally, the quality of the generated HC-LC pairs was independent from the order of generation of the HC and the LC, i.e., prompting with [HC] and [LC].

**Fig 7:**
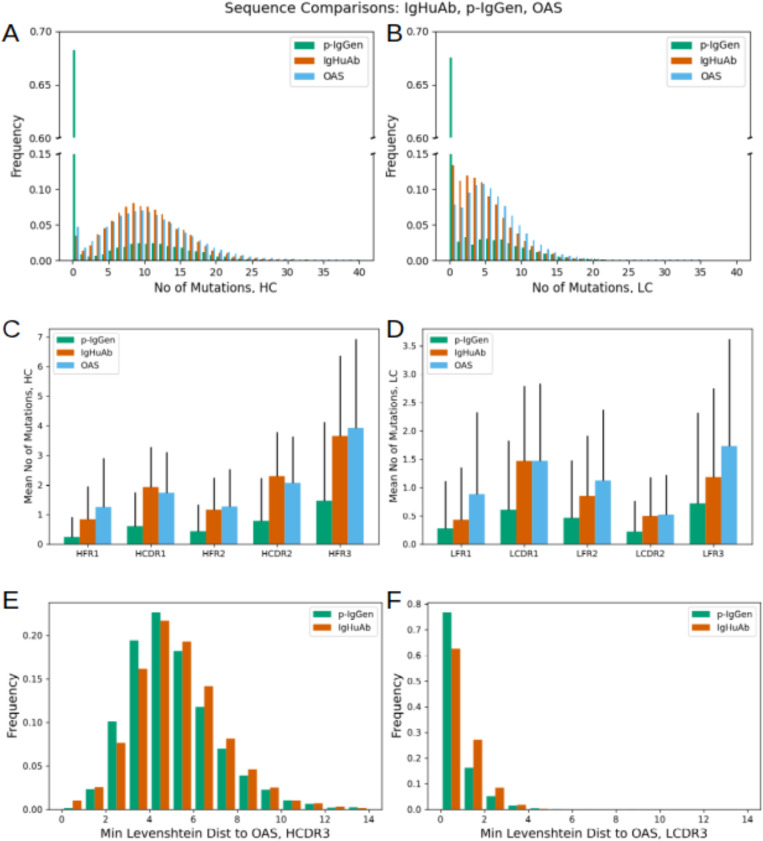
Number of Mutations of the IgHuAb generated HC and LC (in red) vs Number of Mutations of OAS HC and LC (in blue) vs Number of Mutations of the p-IgGen generated HC and LC (in green). (A) Number of Mutations (up to CDR3) for IgHuAb(mean 9.86, std 5.35), p-IgGen(mean 3.53, std 5.93) and OAS(mean 10.24, std 6.38), HC, (B) Number of Mutations (up to CDR3) for IgHuAb(mean 4.42, std 3.86), p-IgGen(mean 2.28, std 4.25) and OAS(mean 5.72, std 4.69), LC, (C) Mean Number of Mutations per antibody region for IgHuAb, p-IgGen and OAS, HC, (D) Mean Number of Mutations per antibody region for IgHuAb, p-IgGen and OAS, LC, (E) Minimum Levenshtein Distance to OAS, HCDR3: IgHuAb(mean 4.77,std 2.13), p-IgGen(mean 4.58, std 2.04), (F) Minimum Levenshtein Distance to OAS, LCDR3: IgHuAb(mean 0.50, std 0.75), p-IgGen(mean 0.33, std 0.70).

**Fig 8:**
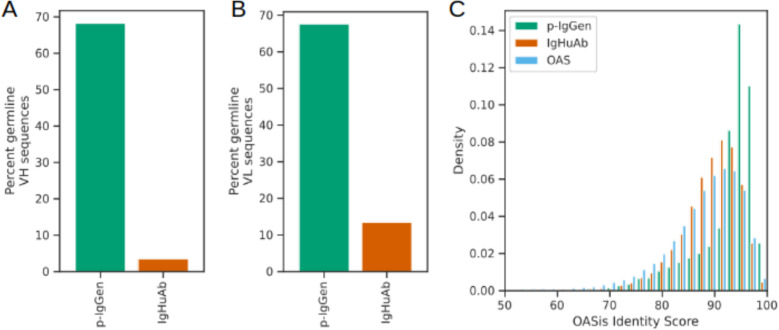
Percent germline sequences (up to CDR3) for the HCs of IgHuAb(in red) and p-IgGen(in green); percent germline sequences (up to CDR3) for the LCs of IgHuAb(in red) and p-IgGen(in green); OASis Identity score comparison of OAS(in blue). IgHuAb(in red) and p-IgGen(in green), (A) Percent germline VH sequences for IgHuAb(3.45) and p-IgGen(68.30), (B) Percent germline VL sequences for IgHuAb(13.44) and p-IgGen(67.6), (C) OASis BioPhi Identity Scores for OAS(mean 87.42, std 8.30), IgHuAb(mean 89.00, std 5.92) and p-IgGen(mean 92.3, std 5.77).

To compare IgHuAb with an existing paired antibody HC-LC generating decoder models, we generated a dataset of 10,000 sequence pairs using p-IgGen [18] (**Fig 7**). In terms of number of mutations (up to and including FWR3), IgHuAb (mean 9.86, std 5.35) more closely recovered the OAS distribution for HCs (mean 10.24, std 6.38), compared to p-IgGen (mean 3.53, std 5.93); similarly for LCs: IgHuAb (mean 4.42 std 3.86), OAS (mean 5.72, std 4.69) and p-IgGen (mean 2.28, std 4.25) (**Fig 7A** and **7B**). The same trend was observed for different sub-regions of the antibody sequence (**Fig 7C** and **7D**) for both HCs and LCs. Notably, p-IgGen generated a larger number of germline (up to and including FWR3) sequences (**Fig 8A** and **8B**). This could also contribute to the larger OASis Identity scores (mean 92.48, std 5.77) achieved by p-IgGen (**Fig 8C**).

**Fig 9:**
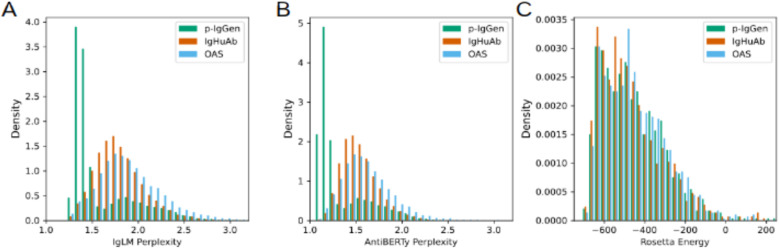
FLAb Benchmarking- OAS(in blue ). IgHuAb(in red) and p-IgGen(in green); IgLM Perplexity, AntiBerty Perplexity and Rosetta Energy, (A) IgLM Perplexity: OAS(mean 1.92, std 0.42), IgHuAb(mean 1.80, std 0.27) and p-IgGen(mean 1.59, std 0.35), (B) AntiBERTy Perplexity: OAS(mean 1.62, std 0.31), IgHuAb(mean 1.54, std 0.21) and p-IgGen(mean 1.33, std 0.28). (C) Rosetta Energy: OAS(mean -449.42, std 150.80), IgHuAb(mean -464.38, std 167.10) and p-IgGen(mean -455.00, std 154.78).

Additionally, we evaluated sequences generated by IgHuAb and p-IgGen, as well as 10,000 randomly selected sequences from OAS, using FLAb (Fitness Landscape for Antibodies), a software package for assessing antibody fitness using deep learning models [19]. From FLAb, we employed several metrics to score antibody sequences (**Fig 9**), including perplexities calculated by the language models IgLM [14] and AntiBERTy [21], as well as the Rosetta Energy [22] for predicted structures of a random subsample of 1,000 antibody sequence pairs for each of the three datasets. p-IgGen (mean 1.59, std 0.35) had the lowest IgLM perplexity, followed by IgHuAb (mean 1.80, std 0.27) and OAS (mean 1.92, std 0.42) with the highest perplexity. Analogous results were observed for the AntiBERTy perplexity: p-IgGen (mean 1.33, std 0.28), IgHuAb (mean 1.54, std 0.21) and OAS (mean 1.62, std 0.31). While lower perplexity scores may correlate with increased antibody fitness in certain contexts [16], these results indicate that IgHuAb more closely recapitulates the distributions in the OAS in comparison with p-IgGen, with the lower perplexity for p-IgGen potentially related to the lower degree of SHM observed for those antibody sequences. Separately, the Rosetta Energy scores were better for IgHuAb (mean -464.38, std 167.10), compared to OAS (mean -449.42, std 150.80), and p-IgGen (mean -455.00, std 154.78).

**Fig 10:**
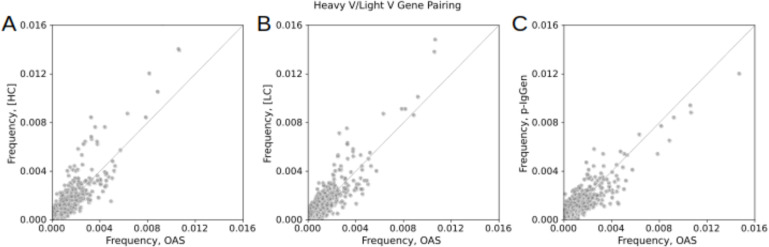
Heavy V/Light V Gene Pairing: OAS vs IgHuAb [HC] prompt generated, IgHuAb [LC] prompt generated, and p-IgGen. (A) Heavy V/Light V Gene Pairing: OAS vs IgHuAb [HC] prompt generated (Pearson correlation 0.913, p<0.0001), (B) Heavy V/Light V Gene Pairing: OAS vs IgHuAb [LC] prompt generated (Pearson correlation 0.920, p<0.0001), (C) Heavy V/Light V Gene Pairing: OAS vs p-IgGen generated (Pearson correlation 0.904, p<0.0001).

To assess the ability of IgHuAb to generate paired antibody sequences that reproduce IGHV/IGLV pairings observed in nature, we compared the distributions of the frequencies of heavy (IGHV) and light (IGLV) chain germline gene pairing for the three sets of antibodies (**Figs 10** and S1-S4). Indeed, a significant correlation was observed between the frequencies with which different IGHV/IGLV pairings were observed for the [HC] prompt antibodies and OAS antibodies (**Fig 10A**) (Pearson correlation: 0.913, p-value<0.0001). Similarly, the IGHV/IGLV frequencies correlated between [LC] prompt antibodies and OAS antibodies (**Fig 10B**) (Pearson correlation: 0.920, p-value<0.0001). p-IgGen antibodies and OAS antibodies had a similar degree of correlation (**Fig 10C**) (Pearson correlation: 0.904, p-value<0.0001). These results suggest that IgHuAb, irrespective of the order in which the HC and LC of the antibodies are generated by the algorithm, reproduces the frequencies of pairing between heavy and light chains in the underlying distribution of naturally occurring human antibodies.

**Fig 11:**
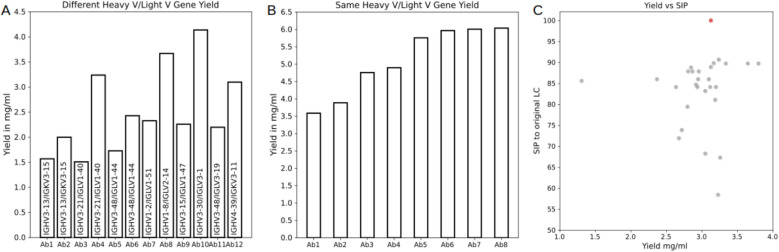
Antibody Expression Yield for Selected Antibodies from SynAbLib. (A) Yield from generated antibodies with diverse VH/VL combinations, (B) Yield from generated antibodies with the same VH/VL combination and high CDRH3/L3 identity, (C) Yield from generated antibodies with the same heavy chain (y-axis) vs sequence identity percent to the native LC sequence (x-axis). The original native antibody heavy-light chain pairing is shown in red.

To assess the ability of IgHuAb to generate paired heavy-light chain sequences that are compatible and can produce viable monoclonal antibodies, we selected sets of IgHuAb-generated paired heavy-light chain sequences to validate experimentally, specifically focusing on expression yield as a readout (**Fig 11**). First, we selected a set of 12 antibodies that represented multiple diverse combinations of VH and VL germline genes (**Fig 11A**). While there were up to ~3-fold differences in expression yield, including for antibodies utilizing the same VH/VL combination, all antibodies showed robust yields, with at least ~1.5 mg/ml (**Fig 11A**). Next, we selected a set of 8 antibodies that utilized the same VH/VL combination, and that had at least 60% CDRH3 sequence identity and at least 90% CDRL3 sequence identity to another antibody in this set (**Fig 11B**). The yields for all antibodies were again robust, at over at least 3 mg/ml and all within less than a 2-fold difference (**Fig 11B**). Finally, we generated and selected a set of 27 antibodies that all used the exact same antibody heavy chain sequence (from an existing naturally occurring antibody) but that were paired with diverse light chain sequences (i.e., IgHuAb was prompted with the full antibody heavy chain sequence, and generated different light chain sequences to pair with the prompt) (**Fig 11C**). As with the other antibody sets, all antibodies exhibited robust yields, with all but one of the antibodies having a yield of ~2.5 mg/ml or more, and several antibodies showing somewhat greater yields than the native heavy-light chain pairing for the original antibody (**Fig 11C**). Importantly, antibody yield did not correlate with the distance of the IgHuAb-generated light chains from the original native light chain sequence (p=0.15, Spearman) (**Fig 11C**). Together, these results indicate that IgHuAb can successfully generate well-expressing monoclonal antibodies with compatible pairing of heavy and light chains, for diverse VH/VL combinations.

## Discussion

Screening the repertoires of naturally occurring antibodies in humans have served as a source for therapeutic and vaccine discovery and have provided important insights into the fundamental rules of antibody sequence diversification and antibody-antigen recognition. However, experimental antibody repertoire screening is associated with high costs, requirements for access to specific samples and specialized instrumentation, and is difficult to scale. In contrast, computational methods for antibody sequence generation can present an efficient, scalable, and generalizable alternative to experimental antibody sequencing approaches. In this manuscript, we present IgHuAb, a large language model algorithm for *de novo* antibody sequence generation for paired heavy and light chains. Using IgHuAb, we generated SynAbLib, a synthetic library of antibody sequences. Analysis of the SynAbLib sequences confirmed that they reproduce characteristic features of naturally occurring antibodies at the population level, including CDR3 length distributions and frequency of specific VH/VL pairing. Our results also indicated that the generated sequences are human-like, can exhibit notable somatic hypermutation levels (albeit not as extreme as some naturally occurring antibodies), and afford additional diversity in sequence space compared to naturally occurring antibodies.

The SynAbLib library is both scalable and specializable. Additional antibody sequence can be generated at a low computational cost (~4 seconds per antibody sequence on a Nvidia A6000 GPU processor) should additional exploration of antibody sequence space be desired. Further, the sequence generation process is flexible: we generated sequences either starting with the heavy chain ([HC] prompt) or the light chain ([LC] prompt). Both of these cases require no part of the antibody sequence to be provided as input, and are therefore completely *de novo*. However, IgHuAb can be applied to generate any portion of the paired antibody sequence as input, which can be of value in cases such as biased sequence generation toward specific germline genes, or the identification of alternative pairings for a given input heavy or light chain sequence, respectively. These features of the IgHuAb algorithm, and the expandable SynAbLib library, make them a valuable tool for studying human antibody repertoires and for antibody discovery in a wide range of settings and applications.

## Methods and materials

### Fine-tuning, paired antibody sequence generation and quality control

The 430,000 paired human antibody sequences were curated from the OAS Paired [8] and PlaAbDab [23] public databases (both accessed in Feb 2024), and the repeating pairs of sequences were removed. Germline genes and CDRs were assigned by ANARCI (implemented as AbNumber in Python) [24] with the *allowed_species=’human’* option. The non-redundant training data was clustered with Linclust [25] and clusters with combined ~10% of the sequences were set aside for model testing.

For domain adaptation fine-tuning, the ProGen2-OAS protein language model was selected. ProGen2-OAS is a 764 million parameters decoder model with 27 transformer blocks, trained on unpaired heavy and light chain sequences from OAS. Two new special tokens were added to the ProGen2 tokenizer: *[HC]* and *[LC].* Each antibody sequence pair ***i*** from the training dataset was passed to the model twice, once as:

**<|*bos*> *[HC]* [heavy chain sequence *i*} *[LC]* [light chain sequence *i*} *<|eos|>***

and once as:

**<|*bos*> *[LC]* [light chain sequence *i*} *[HC]* [heavy chain sequence *i*} *<|eos|>***

not in consecutive order.

The model was fine-tuned using *HuggingFace Trainer* (Adam optimizer, lr= 1x10^-5^, linear learning rate scheduler, training batch size=2) for two epochs on 4 Tesla V100 GPUs. Different numbers of frozen layers(FL) were attempted (0, 4, 8) with similar evaluation losses (initial loss ~19.7132). In all cases the loss decreased during the first epoch suggesting that the model recognizes the domain adaptation task of generating paired sequences.

 Loss, Epoch 1 Loss, Epoch 2

 FL=0 0.082396 0.093879

 FL=4 0.082687 0.095155

 FL=8 0.082717 0.095282

In order to select a checkpoint, we used the quality of the output (viable human (as determined by Abnumber) pairs of sequences) by generating 1000 HC-LC pairs of sequences for both [HC] and [LC] prompts.

 Viable Pairs(FL=0) Viable Pairs(FL=4) Viable Pairs(FL=8)

Epoch 1 [HC], 1000 [HC], 938 [HC], 46

 [LC], 1000 [LC], 1000 [LC], 0

Epoch 2 [HC], 1000 [HC], 962 [HC], 2

 [LC], 1000 [LC], 249 [LC], 0

Based on this, and the quality of the output, for antibody generation, we selected the unfrozen model checkpoint (FL=0) saved after epoch 1 (IgHuAb). The paired antibody sequences were generated with maximal sequence length of 250 amino acids, probability threshold of 0.9 and temperature of 1.2. For generation of the synthetic library SynAbLib, we used several different prompts: 500,000 sequences with prompt *[HC]*, and 500,000 with *[LC]*.

Each generated output was filtered by selecting the sequence up to the first <|pad|> token, then it was separated into two by using the *[HC]* and the *[LC]* tokens, yielding two sequences: one corresponding to the generated antibody heavy chain and one – to the antibody light chain. All the filtered generated Ab pairs were additionally processed by ANARCI with the *allowed_species=’human’* option and the IMGT numbering scheme, assigning framework regions, CDRs and germline V and J genes for both the heavy and the light chain. All redundant pairs were removed. Finally, mutational distance to germline variable genes (ANARCI), and humanness (BioPhi OASis) was estimated.

### Antibody expression and purification

Antibodies were produced by GenScript with the TurboCHO high throughput platform.

## Supporting information

S1 Fig Frequencies of Heavy V/ Light V Gene Combinations, OAS. (TIFF)

S2 Fig Frequencies of Heavy V/ Light V Gene Combinations, [HC] prompt. (TIFF)

S3 Fig Frequencies of Heavy V/ Light V Gene Combinations, [LC] prompt. (TIFF)

S4 Fig Frequencies of Heavy V/ Light V Gene Combinations, p-IgGen. (TIFF)
