# Supplementary figures and images for: An expandable synthetic library of human paired antibody sequences
Source: PLoS Comput Biol. 2025 Apr 21;21(4):e1012932. doi: 10.1371/journal.pcbi.1012932 (PMC12011268; doi:10.1371/journal.pcbi.1012932)

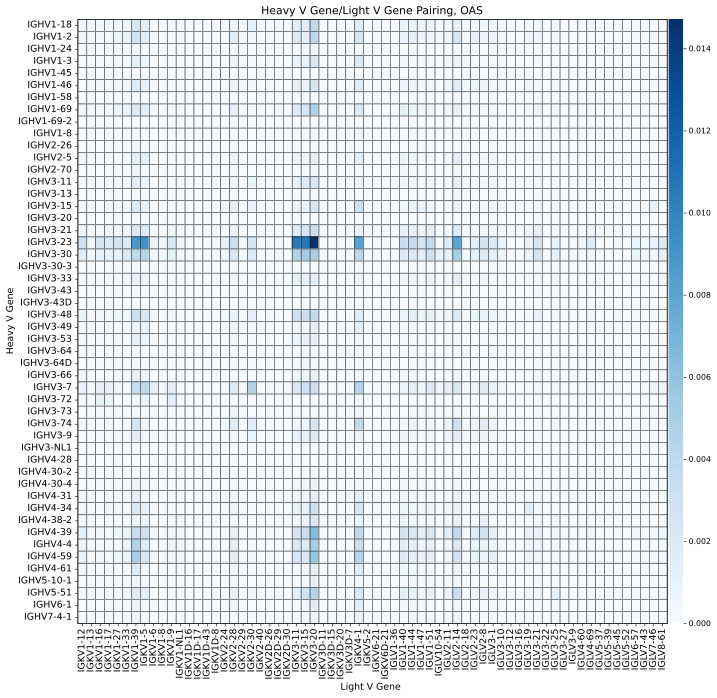

Supplement: S1 Fig — (TIFF) [file pcbi.1012932.s001.tiff]

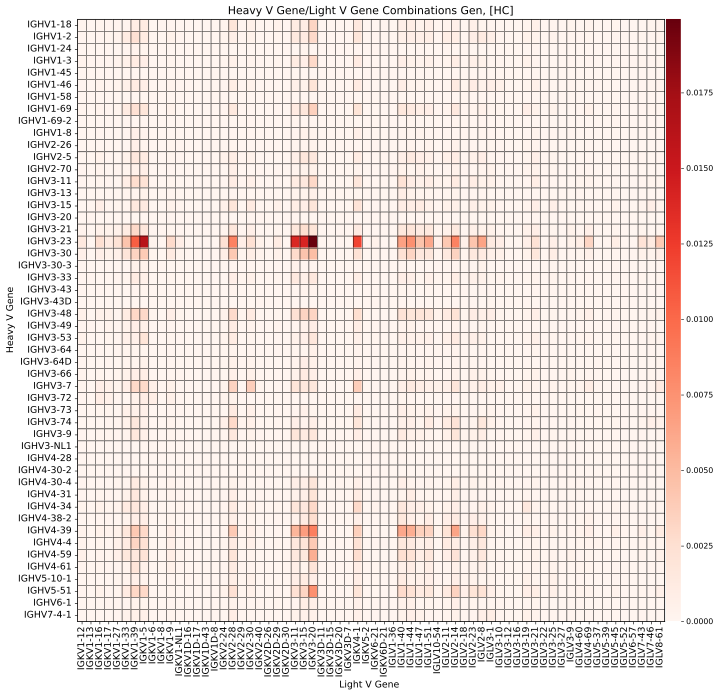

Supplement: S2 Fig — (TIFF) [file pcbi.1012932.s002.tiff]

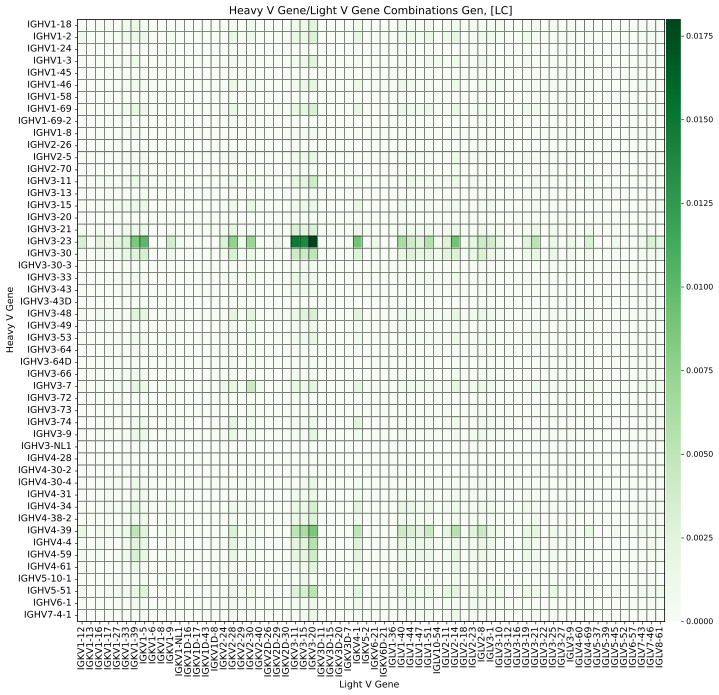

Supplement: S3 Fig — (TIFF) [file pcbi.1012932.s003.tiff]

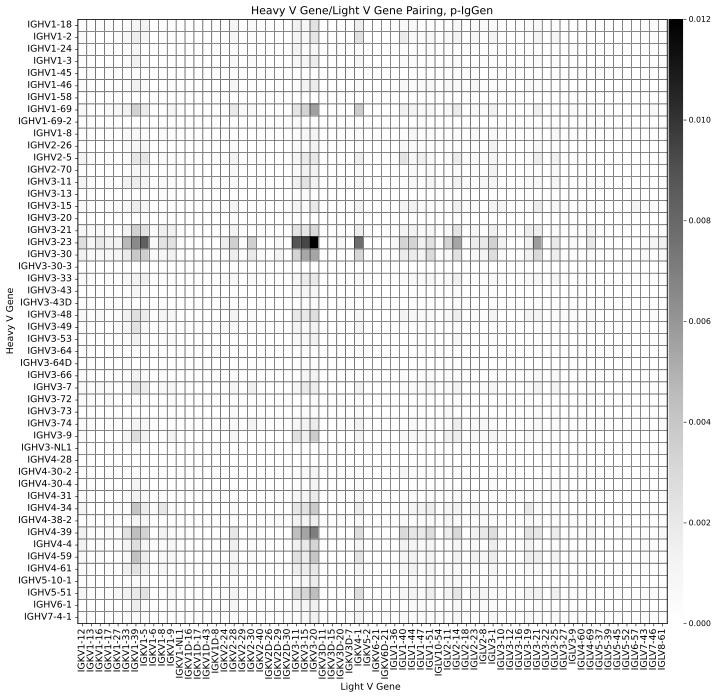

Supplement: S4 Fig — (TIFF) [file pcbi.1012932.s004.tiff]
